# Supplementary material for: Single Nucleotide Polymorphisms in Genes Encoding Toll-Like Receptors 7 and 8 and Their Association with Proviral Load of SRLVs in Goats of Polish Carpathian Breed
Source: Animals (Basel). 2021 Jun 26;11(7):1908. doi: 10.3390/ani11071908 (PMC8300119; doi:10.3390/ani11071908)
Supplement: Supplementary file 1 [file animals-11-01908-s001.zip › Supplementary Figure S1. The exact TLR7 SNPs localization based on transcript and genomic sequence..pdf]

Gene: TLR7 ENSCHIG00000021012
Transcript: TLR7-201 ENSCHIT00000031299.1
Genome assembly: ARS1 (GCA_001704415.1) Ensembl

<u>cDNA sequence with SNP localization:</u>

```
  1 TCTTTGCACTTGGAAAGTTCACGACTGGCAGCCTGCAGTCTCCATGACTTAAGTAGGTTT    60
    ............................................................
    ............................................................
          LWLT01000021.1_50718645_C/T 5'UTR variant

 61 GCCACAGCTCATCTCTTCACCTCAGAAGACTCCAGACCTGGACTCACTCCATGCAGCAAA   120
    ............................................................
    ............................................................

121 GAAAGTTGATGCTACTGGGTCCATCTCAGGCTGATCTTGACACCTTCCATGTTCTGCTCT   180
    ............................................................
    ............................................................

181 CTTCAGCCAGACTTGTCTGTTTCATTTTGGAAGAAGACTAAAAATGGTGTTTCCAATGTG   240
    ...........................................ATGGTGTTTCCAATGTG    17
    ...........................................-M--V--F--P--M--W     6

241 GACACTGAAGAGACAGTTTCCTATCCTTTTTAACATGATCCTAATTTCTGAACTCCTTGG   300
 18 GACACTGAAGAGACAGTTTCCTATCCTTTTTAACATGATCCTAATTTCTGAACTCCTTGG    77
  6 --T--L--K--R--Q--F--P--I--L--F--N--M--I--L--I--S--E--L--L--G    26

301 GGCTAGATGGTTTCCTAAAACTCTGCCCTGTGATGTCACTCTGGATGCTCCAAATGCCCA   360
 78 GGCTAGATGGTTTCCTAAAACTCTGCCCTGTGATGTCACTCTGGATGCTCCAAATGCCCA   137
 26 --A--R--W--F--P--K--T--L--P--C--D--V--T--L--D--A--P--N--A--H    46

361 TGTGATTGTGGACTGCACAGACAAACATTTGACAGAAATTCCTGGAGGTATTCCTGCCAA   420
138 TGTGATTGTGGACTGCACAGACAAACATTTGACAGAAATTCCTGGAGGTATTCCTGCCAA   197
 46 --V--I--V--D--C--T--D--K--H--L--T--E--I--P--G--G--I--P--A--N    66

421 TGCCACCAACCTCACCCTCACCATTAACCACATAGCAGGCATCTCTCCAGCCTCCTTCCA   480
198 TGCCACCAACCTCACCCTCACCATTAACCACATAGCAGGCATCTCTCCAGCCTCCTTCCA   257
 66 --A--T--N--L--T--L--T--I--N--H--I--A--G--I--S--P--A--S--F--H    86

481 CCGGCTGGACCATCTGGTGGAGATCGATTTCAGGTGCAACTGCATACCTGTTCGACTGGG   540
258 CCGGCTGGACCATCTGGTGGAGATCGATTTCAGGTGCAACTGCATACCTGTTCGACTGGG   317
 86 --R--L--D--H--L--V--E--I--D--F--R--C--N--C--I--P--V--R--L--G   106

541 GCCAAAAGACAACGTGTGCACCAAAAGGCTACAGATTAAACCCAACAGCTTTAGCAAACT   600
318 GCCAAAAGACAACGTGTGCACCAAAAGGCTACAGATTAAACCCAACAGCTTTAGCAAACT   377
106 --P--K--D--N--V--C--T--K--R--L--Q--I--K--P--N--S--F--S--K--L   126

601 CACGTATTTAAAATCTCTTTACCTGGATGGAAACCAGCTTCTAGAAATACCTCAGGATCT   660
378 CACGTATTTAAAATCTCTTTACCTGGATGGAAACCAGCTTCTAGAAATACCTCAGGATCT   437
126 --T--Y--L--K--S--L--Y--L--D--G--N--Q--L--L--E--I--P--Q--D--L   146
```

```
661 TCCTCCCAGCTTACAGCTGCTGAGCCTGGAGGCCAACAACATCTTCTTGATCATGAAGGA 720
438 TCCTCCCAGCTTACAGCTGCTGAGCCTGGAGGCCAACAACATCTTCTTGATCATGAAGGA 497
146 --P--P--S--L--Q--L--L--S--L--E--A--N--N--I--F--L--I--M--K--E 166

721 GAATCTAACAGAACTGGCCAACCTAGAAATACTCTACCTGGGCCAAAACTGTTACTATCG 780
498 GAATCTAACAGAACTGGCCAACCTAGAAATACTCTACCTGGGCCAAAACTGTTACTATCG 557
166 --N--L--T--E--L--A--N--L--E--I--L--Y--L--G--Q--N--C--Y--Y--R 186

781 TAACCCTTGTAATGTTTCATTTACTATCGAAAAAGATGCTTTCCTAAATATGAGAAATTT 840
558 TAACCCTTGTAATGTTTCATTTACTATCGAAAAAGATGCTTTCCTAAATATGAGAAATTT 617
186 --N--P--C--N--V--S--F--T--I--E--K--D--A--F--L--N--M--R--N--L 206

841 AAAATTGCTCTCCCTAAAAGATAACAATATCTCAGCTGTCCCCACTGTTTTGCCATCTAG 900
618 AAAATTGCTCTCCCTAAAAGATAACAATATCTCAGCTGTCCCCACTGTTTTGCCATCTAG 677
206 --K--L--L--S--L--K--D--N--N--I--S--A--V--P--T--V--L--P--S--S 226

901 TTTGACAGAACTCTATCTTTACAATAACATCATTACAAAAATCCAAGAAGATGATTTTAA 960
678 TTTGACAGAACTCTATCTTTACAATAACATCATTACAAAAATCCAAGAAGATGATTTTAA 737
226 --L--T--E--L--Y--L--Y--N--N--I--I--T--K--I--Q--E--D--D--F--N 246

961 TAACCTCAGTCAACTACAAGTTCTTGATCTGAGTGGAAATTGCCCTCGTTGTTATAATGT 1020
738 TAACCTCAGTCAACTACAAGTTCTTGATCTGAGTGGAAATTGCCCTCGTTGTTATAATGT 797
246 --N--L--S--Q--L--Q--V--L--D--L--S--G--N--C--P--R--C--Y--N--V 266

1021 TCCATTTCCTTGCACACCCTGTGAAAATAATTCTCCCCTACAGATTGATCTAAATGCTTT 1080
798 TCCATTTCCTTGCACACCCTGTGAAAATAATTCTCCCCTACAGATTGATCTAAATGCTTT 857
266 --P--F--P--C--T--P--C--E--N--N--S--P--L--Q--I--D--L--N--A--F 286

1081 TGATGCATTGACAGAATTGCAAGTCTTACGCCTACACAGTAACTCTCTTCAGCATGTGCC 1140
858 TGATGCATTGACAGAATTGCAAGTCTTACGCCTACACAGTAACTCTCTTCAGCATGTGCC 917
286 --D--A--L--T--E--L--Q--V--L--R--L--H--S--N--S--L--Q--H--V--P 306

1141 CCAAAGATGGTTTAAAAACATTAACAAACTTAAAGAACTAGATCTTTCCCAAAACTTCTT 1200
918 CCAAAGATGGTTTAAAAACATTAACAAACTTAAAGAACTAGATCTTTCCCAAAACTTCTT 977
306 --Q--R--W--F--K--N--I--N--K--L--K--E--L--D--L--S--Q--N--F--L 326

1201 GGCCAAAGAAATTGGGGATGCCAAATTTTTACATCTTCTTCATAACCTTGTCAACTTGGA 1260
978 GGCCAAAGAAATTGGGGATGCCAAATTTTTACATCTTCTTCATAACCTTGTCAACTTGGA 1037
326 --A--K--E--I--G--D--A--K--F--L--H--L--L--H--N--L--V--N--L--D 346

1261 TCTGTCTTTCAATTATGATCTTCAGGTCTACCATGCAGTTATAAATCTATCAGATGCATT 1320
1038 TCTGTCTTTCAATTATGATCTTCAGGTCTACCATGCAGTTATAAATCTATCAGATGCATT 1097
346 --L--S--F--N--Y--D--L--Q--V--Y--H--A--V--I--N--L--S--D--A--F 366

1321 TTCTTCACTGAAAAAATTGAAAGTTTTGCGAATCAAAGGCTACGTCTTTAAAGAGCTGAA 1380
1098 TTCTTCACTGAAAAAATTGAAAGTTTTGCGAATCAAAGGCTACGTCTTTAAAGAGCTGAA 1157
366 --S--S--L--K--K--L--K--V--L--R--I--K--G--Y--V--F--K--E--L--N 386

1381 TAGTTTAAACCTCTTCCCATTATGTAATCTTCCCAGTCTTGAAGTTCTTGATCTTGGCAC 1440
1158 TAGTTTAAACCTCTTCCCATTATGTAATCTTCCCAGTCTTGAAGTTCTTGATCTTGGCAC 1217
386 --S--L--N--L--F--P--L--C--N--L--P--S--L--E--V--L--D--L--G--T 406
```

```
1441 TAACTTTATTAAAATTGCTGACCTCAGCATATTTAAACAATTTAAAACATTGAAATTCAT 1500
1218 TAACTTTATTAAAATTGCTGACCTCAGCATATTTAAACAATTTAAAACATTGAAATTCAT 1277
 406 --N--F--I--K--I--A--D--L--S--I--F--K--Q--F--K--T--L--K--F--I 426

1501 TGATCTTTCAGTGAATAAAATATCACCTTCGGGAGATTCAACTGAAGGTGGTTTCTGCTC 1560
1278 TGATCTTTCAGTGAATAAAATATCACCTTCGGGAGATTCAACTGAAGGTGGTTTCTGCTC 1337
 426 --D--L--S--V--N--K--I--S--P--S--G--D--S--T--E--G--G--F--C--S 446

1561 TAACATGAGAACTTCTGTAGAAGGCCATGGGCCCCAGGTCCTTGAAGCATTGCATTATTT 1620
1338 TAACATGAGAACTTCTGTAGAAGGCCATGGGCCCCAGGTCCTTGAAGCATTGCATTATTT 1397
 446 --N--M--R--T--S--V--E--G--H--G--P--Q--V--L--E--A--L--H--Y--F 466

1621 CAGATATGATGAGTATGCAAGGAGTTGCCGGTCCAAGAGCAAAGAGCCTCCTTCTTTCTT 1680
1398 CAGATATGATGAGTATGCAAGGAGTTGCCGGTCCAAGAGCAAAGAGCCTCCTTCTTTCTT 1457
 466 --R--Y--D--E--Y--A--R--S--C--R--S--K--S--K--E--P--P--S--F--L 486

1681 ACCTCTTAATGAAGATTGTTATATGTATGGGCAGACCTTGGACCTTAGTAGAAATAATAT 1740
1458 ACCTCTTAATGAAGATTGTTATATGTATGGGCAGACCTTGGACCTTAGTAGAAATAATAT 1517
 486 --P--L--N--E--D--C--Y--M--Y--G--Q--T--L--D--L--S--R--N--N--I 506

1741 ATTTTTTATCAAGTCTTCTGATTTCCAGCATCTTTCTTTCCTCAAATGCCTAAACTTATC 1800
1518 ATTTTTTATCAAGTCTTCTGATTTCCAGCATCTTTCTTTCCTCAAATGCCTAAACTTATC 1577
 506 --F--F--I--K--S--S--D--F--Q--H--L--S--F--L--K--C--L--N--L--S 526

1801 AGGAAATAGCATTAGCCAGACGCTTAATGGAAGTGAATTTCAGCCTTTAGTGGAGTTGAA 1860
1578 AGGAAATAGCATTAGCCAGACGCTTAATGGAAGTGAATTTCAGCCTTTAGTGGAGTTGAA 1637
 526 --G--N--S--I--S--Q--T--L--N--G--S--E--F--Q--P--L--V--E--L--K 546

1861 ATATTTGGACTTCTCTAACAATCGGCTTGATTTACTCTACTCAACGGCATTTGAGGAGCT 1920
1638 ATATTTGGACTTCTCTAACAATCGGCTTGATTTACTCTACTCAACGGCATTTGAGGAGCT 1697
 546 --Y--L--D--F--S--N--N--R--L--D--L--L--Y--S--T--A--F--E--E--L 566

1921 GCACAACCTGGAAGTCCTAGATATAAGTAGTAACAGCCATTATTTTCAATCAGAAGGAAT 1980
1698 GCACAACCTGGAAGTCCTAGATATAAGTAGTAACAGCCATTATTTTCAATCAGAAGGAAT 1757
 566 --H--N--L--E--V--L--D--I--S--S--N--S--H--Y--F--Q--S--E--G--I 586

1981 TACTCACATGCTAAATTTTACCAAGAACCTCAAGGTTCTGAAGAAACTGATGATGAACTA 2040
1758 TACTCACATGCTAAATTTTACCAAGAACCTCAAGGTTCTGAAGAAACTGATGATGAACTA 1817
 586 --T--H--M--L--N--F--T--K--N--L--K--V--L--K--K--L--M--M--N--Y 606

2041 TAATGACATCGCTACCTCCACCAGCAGGACCATGGAGAGTGAATCTCTTCAAATCCTGGA 2100
1818 TAATGACATCGCTACCTCCACCAGCAGGACCATGGAGAGTGAATCTCTTCAAATCCTGGA 1877
 606 --N--D--I--A--T--S--T--S--R--T--M--E--S--E--S--L--Q--I--L--E 626

2101 GTTCAGAGGCAACCATTTGGATATTTTATGGAGAGATGGTGATAACAGATACTTAAAATT 2160
1878 GTTCAGAGGCAACCATTTGGATATTTTATGGAGAGATGGTGATAACAGATACTTAAAATT 1937
 626 --F--R--G--N--H--L--D--I--L--W--R--D--G--D--N--R--Y--L--K--F 646

2161 CTTCAAGAATCTGCTAAACTTAGAAGAGCTAGACATCTCTGAAAATTCTCTGAGTTTCTT 2220
1938 CTTCAAGAATCTGCTAAACTTAGAAGAGCTAGACATCTCTGAAAATTCTCTGAGTTTCTT 1997
 646 --F--K--N--L--L--N--L--E--E--L--D--I--S--E--N--S--L--S--F--L 666
```

```
2221 ACCTTTGGGAGTTTTTGACAGTATGCCTCCAAATCTAAAGACTCTCTCCTTGGCCAAAAA   2280
1998 ACCTTTGGGAGTTTTTGACAGTATGCCTCCAAATCTAAAGACTCTCTCCTTGGCCAAAAA   2057
 666 --P--L--G--V--F--D--S--M--P--P--N--L--K--T--L--S--L--A--K--N    686

2281 TGGGCTCAAGTCTTTCAGTTGGAAAGGACTACAGAGTCTGAAGAATCTAGAAACTTTGGA   2340
2058 TGGGCTCAAGTCTTTCAGTTGGAAAGGACTACAGAGTCTGAAGAATCTAGAAACTTTGGA   2117
 686 --G--L--K--S--F--S--W--K--G--L--Q--S--L--K--N--L--E--T--L--D    706
```

LWLT01000021.1_50703293_T/C synonymous variant

```
2341 CCTCAGCTTCAACCAGCTGAAGACTGTCCCTGAGAGATTATCCAACTGTTCCCGCAGCCT   2400
2118 CCTCAGCTTCAACCAGCTGAAGACTGTCCCTGAGAGATTATCCAACTGTTCCCGCAGCCT   2177
 706 --L--S--F--N--Q--L--K--T--V--P--E--R--L--S--N--C--S--R--S--L    726

2401 CAAGAAACTCATACTTAAGAATAATCAAATCAGGTGCCTGACAAAGTATTTTCTCCAAGG   2460
2178 CAAGAAACTCATACTTAAGAATAATCAAATCAGGTGCCTGACAAAGTATTTTCTCCAAGG   2237
 726 --K--K--L--I--L--K--N--N--Q--I--R--C--L--T--K--Y--F--L--Q--G    746

2461 TGCTTTCCAGTTGCGACATCTGGACCTCAGCTCAAATAAAATTCAGGTTATCCAAAAGAC   2520
2238 TGCTTTCCAGTTGCGACATCTGGACCTCAGCTCAAATAAAATTCAGGTTATCCAAAAGAC   2297
 746 --A--F--Q--L--R--H--L--D--L--S--S--N--K--I--Q--V--I--Q--K--T    766

2521 GAGTTTTCCAGAAAACGTCCTCAACAATCTAAACATTTTGTTTTTGCATCACAATCGATT   2580
2298 GAGTTTTCCAGAAAACGTCCTCAACAATCTAAACATTTTGTTTTTGCATCACAATCGATT   2357
 766 --S--F--P--E--N--V--L--N--N--L--N--I--L--F--L--H--H--N--R--F    786

2581 TCTGTGCAACTGTGATGCTGTGTGGTTTGTCTGGTGGGTTAACCATACCGAGGTGACTAT   2640
2358 TCTGTGCAACTGTGATGCTGTGTGGTTTGTCTGGTGGGTTAACCATACCGAGGTGACTAT   2417
 786 --L--C--N--C--D--A--V--W--F--V--W--W--V--N--H--T--E--V--T--I    806

2641 TCCTTACTTGGCCACAGATGTGACTTGCATGGGACCAGGAGCACACAAGGGCCAGAGTGT   2700
2418 TCCTTACTTGGCCACAGATGTGACTTGCATGGGACCAGGAGCACACAAGGGCCAGAGTGT   2477
 806 --P--Y--L--A--T--D--V--T--C--M--G--P--G--A--H--K--G--Q--S--V    826

2701 GGTCTCTCTGGATCTATATACCTGTGAGTTAGATTTGACTAACTTCATCCTGTTCTCACT   2760
2478 GGTCTCTCTGGATCTATATACCTGTGAGTTAGATTTGACTAACTTCATCCTGTTCTCACT   2537
 826 --V--S--L--D--L--Y--T--C--E--L--D--L--T--N--F--I--L--F--S--L    846

2761 TTCCATATCAGCAATTCTCTCTCTGATGATGATCACAACAGCAAACCATCTCTATTTCTG   2820
2538 TTCCATATCAGCAATTCTCTCTCTGATGATGATCACAACAGCAAACCATCTCTATTTCTG   2597
 846 --S--I--S--A--I--L--S--L--M--M--I--T--T--A--N--H--L--Y--F--W    866

2821 GGATGTGTGGTATAGTTATCATTTCTGTAAAGCCAAAATAAAAGGGTATCGACGTCTGAT   2880
2598 GGATGTGTGGTATAGTTATCATTTCTGTAAAGCCAAAATAAAAGGGTATCGACGTCTGAT   2657
 866 --D--V--W--Y--S--Y--H--F--C--K--A--K--I--K--G--Y--R--R--L--I    886

2881 ATCACCCAATTCTTGCTATGATGCTTTCATTGTATATGACACTAAAGACCCAGCAGTGAC   2940
2658 ATCACCCAATTCTTGCTATGATGCTTTCATTGTATATGACACTAAAGACCCAGCAGTGAC   2717
 886 --S--P--N--S--C--Y--D--A--F--I--V--Y--D--T--K--D--P--A--V--T    906

2941 AGAGTGGGTTTTGGACGAGCTGGTGGCCAAATTGGAAGACCCGAGAGAGAAGTGTTTTAA   3000
2718 AGAGTGGGTTTTGGACGAGCTGGTGGCCAAATTGGAAGACCCGAGAGAGAAGTGTTTTAA   2777
```

```
 906 --E--W--V--L--D--E--L--V--A--K--L--E--D--P--R--E--K--C--F--N   926

3001 TTTATGTCTTGAGGAAAGAGACTGGTTACCAGGGCAGCCTGTTCTGGAAAATCTTTCCCA  3060
2778 TTTATGTCTTGAGGAAAGAGACTGGTTACCAGGGCAGCCTGTTCTGGAAAATCTTTCCCA  2837
 926 --L--C--L--E--E--R--D--W--L--P--G--Q--P--V--L--E--N--L--S--Q   946

3061 GAGCATACAGCTTAGCAAAAAGACAGTGTTTGTGATGACAGACAAGTACGCAAAGACTGA  3120
2838 GAGCATACAGCTTAGCAAAAAGACAGTGTTTGTGATGACAGACAAGTACGCAAAGACTGA  2897
 946 --S--I--Q--L--S--K--K--T--V--F--V--M--T--D--K--Y--A--K--T--E   966

3121 GAATTTTAAGATAGCATTTTACTTATCCCATCAGAGGCTCATGGATGAAAAAGTGGATGT  3180
2898 GAATTTTAAGATAGCATTTTACTTATCCCATCAGAGGCTCATGGATGAAAAAGTGGATGT  2957
 966 --N--F--K--I--A--F--Y--L--S--H--Q--R--L--M--D--E--K--V--D--V   986

3181 AATCATCTTGATATTCCTTGAGAAGCCCCTTCAGAAGTCCAAGTTTCTCCAACTCCGGAA  3240
2958 AATCATCTTGATATTCCTTGAGAAGCCCCTTCAGAAGTCCAAGTTTCTCCAACTCCGGAA  3017
 986 --I--I--L--I--F--L--E--K--P--L--Q--K--S--K--F--L--Q--L--R--K  1006

3241 GAGGCTCTGTGGCAGTTCTGTCCTTGAGTGGCCAACAAACCCACAGGCTCACCCGTACTT  3300
3018 GAGGCTCTGTGGCAGTTCTGTCCTTGAGTGGCCAACAAACCCACAGGCTCACCCGTACTT  3077
1006 --R--L--C--G--S--S--V--L--E--W--P--T--N--P--Q--A--H--P--Y--F  1026

3301 CTGGCAGTGTCTGAAAAATGCCCTGGCCACAGACAATCACGTGACCTACAGTCAGGTGTT  3360
3078 CTGGCAGTGTCTGAAAAATGCCCTGGCCACAGACAATCACGTGACCTACAGTCAGGTGTT  3137
1026 --W--Q--C--L--K--N--A--L--A--T--D--N--H--V--T--Y--S--Q--V--F  1046

3361 CAAAGAGACAGCCTAGCCCTTCTTTGCCGAATGTGACTGCTACCAAGGAGAAGCTCAGCT  3420
3138 CAAAGAGACAGCCTAG............................................  3153
1046 --K--E--T--A--*-............................................  1050

3421 GCCTTGATCGGCTCACCCATGCCTTGCCAGAAGAGTGTTCTAAGATTCTCCAAGCCCTGG  3480
     ............................................................
     ............................................................

3481 GATTGCCCATATTGGAGAGGAGTCACCAATACATAACAAAGGAAGCAGAAAAATGGGATT  3540
     ............................................................
     ............................................................

                     LWLT01000021.1_50702074_T/C 3'UTR variant

3541 TATATAAGCATCAAGTCATCTTTCTCAGCTCTCTCTGTCTCCATTTGCACTTGAGTCTTT  3600
     ............................................................
     ............................................................

3601 GTCTTCTGCCCTTGCATAAAATACTGTTGGGAGAAGGGTGGCAAGTAGAGGATGTGGGAC  3660
     ............................................................
     ............................................................

3661 TTTGATTCTCCTGTAATTGTGATTATTACATATACACACAGTCACCAAGAACTGCACTTC  3720
     ............................................................
     ............................................................
```

3721 TACCCTCAAGAGGCACTGGTATGTATAGAAATAGGGTTAAAAAAAAGGCTCAGAGTCTGC 3780
....
....

3781 TTATATGACATTAAAATGTACCAGTTAATTAGTGGTAAAAATAAAGACACAGTTAACTCC 3840
....
....

3841 TCAGCCAATTGACTCAACCATCTAGTGCCCCGTTCTGTGCAGACCAAGTGCTGGCCCCCA 3900
....
....

3901 GCAGGTGGTACAGCTCAAGTGCTCCTTGCTCTTTCTCTCCTGGGCCTGTTACTGGGTTTT 3960
....
....

3961 TTGGGGAAATAGAAACATCTTTGTCATCGATGGACATAGTCTACTTACAAGTGGGAGAAG 4020
....
....

4021 AATAAAGTCATGCTCTTTACTTTTGATGATAATTTACCTGCTTAAATATGGCTCTGGACT 4080
....
....

4081 AAATGTTGAAATCCTAATCCCCCAGTGTGATGGTGTTGGGAGGTGGGAGGTGGGGTTTTT 4140
....
....

4141 AAGAAGTGGTTAGGTCATGAGACTGGAGCCCTCGTGATGGGATTACTGCCTTAGAAGAAG 4200
....
....

4201 CAGTCAGGGGACTAACTTGCTGTGTTCCCATCGTGTGAGGGTATGGAGAGAGGCTGGCAG 4260
....
....

4261 TCTATAACACACAGGAGGACCCTCAGCAGAACCAGATCATATT**G**ACACTCTGATCTCCTT 4320
....
....

LWLT01000021.1_50701297_C/T

4321 CAGACTGGCTTCCATGCTTCAGACTTGTGAGAAATAAATGTTTGTTCTTTAAGTCATGCA 4380
....
....

4381 GTTTGTGATGTTTTGTTACAGCAGCTGAGCTAAGACAGATTTTTTTAATCTGCATGGCAA 4440
....
....

4441 AGTACTGTTTCCAAAGAAAAGTTGCCTCATCCAATGATCTTTCAAACTGCTTTATTAACT 4500
....

```
     ............................................................

4501 AATACTGTATACTTGTAATTATCTGAAAACTTGATCCATCAATGTCAGACAGCTTTGATG 4560
     ............................................................
     ............................................................

4561 ATAAACTCTAAAGGAGAACCCAAGAGCATATATTTATTTAGAGTTAGCAAGCAGGGATGA 4620
     ............................................................
     ............................................................

4621 CAGTCACATGGATTCCTTTAAATTTGGAGGCTGGGGGAGGAACAGAGGTTTACCACCATT 4680
     ............................................................
     ............................................................

4681 AACACTGAGAAAGCTTCAGTCTTGAGCTTTGGCTTCAGTACTAAAAAAAAAAAAAAAAAA 4740
     ............................................................
     ............................................................

4741 AAAACACCAGAAAGGACCAAGATGTTCTGAAGACACACATACTTTCTAAGTGTCTATAAA 4800
     ............................................................
     ............................................................

4801 TTTGCATGAGTCCAAAGTCTGAAATGTGACCTGTTTGTTTTTATTTCATGAAAGAAGTTA 4860
     ............................................................
     ............................................................

4861 TCTACAGAGGTGTCTGCGCCCTTTGAAAGATAGCTTCAAATCCCAGACTTCAAATTATCT 4920
     ............................................................
     ............................................................

4921 GGGAGATAACTGTGTTTTCTTCATCTATGGAAAAGCCAAAGGTTTTACTCTGAGGATGGC 4980
     ............................................................
     ............................................................

4981 TATATGTATTATTTCCAAAGGACATGTAATCAATAAGGGTCACAAACTCCCAAATTAATC 5040
     ............................................................
     ............................................................

5041 TCTGGAATCAACAGAGCCAGCTGTCTGATAGCCTCGGTAAGCTCGATTGTACTTCATAGG 5100
     ............................................................
     ............................................................

5101 CGCCTACGTGAAAACACAGATAACGCCATAAAAACTGGCTGTTGGCATCATCAATTTCTT 5160
     ............................................................
     ............................................................

5161 CTATTACAACACATAACTATACAAGGTTTAAGTTATTTTGGTGATTTATAAACCATGAAG 5220
     ............................................................
     ............................................................

5221 CACTTGAAGACAAACATCTTCTGAAATGAACCAAGAGGAAGGGAAACTGACAACACAACT 5280
     ............................................................
```

....

5281 CAGAAACCGCAGTGTATTTCAACATGAGGCTGTGCAGCAGTGGTTTGTTGTAGGAAATGA 5340
....
....

5341 CCCAAAGCACAGTAACAGGTGGTTTTCATTTTAATTCCTTTGTATCTTGACAAGTCACTC 5400
....
....

5401 TGTTAATCTCCTGAGGGTCCTCTGTGGAGTCCTATACCATTAGAGCCATTCATAGATGGC 5460
....
....

5461 TCGGCTCGAAAGCCACCAACTCAGGCCACCAGGAGAACATGACTGCTAGGTCCCCTGCCA 5520
....
....

5521 AGCTCTGAGTATGGAAGAGACTCCAGATTGTCCATGGATCCCATGCTACCATGAAGGGGT 5580
....
....

5581 TCCCAACCACTTCCAGCCCTGAACCTGTCCTTCCTCCTCGGATAGAGACTGTACCTTAGC 5640
....
....

5641 ACGACTTGGGCATTTGTAGGATCCAAGCCAAGAGAGGCATTGGGCAG 5687
....
....

Genomic DNA (reverse strand) sequence with SNP and primers localization:

[1]LWLT01000021.1_50718760_G/A promoter variant

[2]LWLT01000021.1_50718645_C/T 5’UTR variant

[3]LWLT01000021.1_ 1: 50718466_C/T intron variant

CTGCCCCTGCTTAGAAACCAGAGTCCCTTTCAGCTGAGCCTAAGAGAGAACAAGGGAAAT
GTAGACATGTCTCAAAGATACCTGTAGAATGACGGTTCTCAGTCAGGGGCCGGGGATGTG
TTACAATGTCTAGAGACATCTGTGATTGTTGAGGGGCAGTGTGGCTGGCATCTCATGGGC
TCAGGTCAGGGTTGCTGCTCAGTGTCTTACAATGCCACAAAGATGTATCTCACCAGAAAT
GGCAGGAGTGCACCAGTTGAGAAACTTCTAGAAAAAAACATCAGCCAGCCAAGTTGCTTG
AATTTAGACATGAGGTTGCCCTGATTG**C**[1]GGTGGTAGAAATAGAAGGAACCTGCTGCTTCT
GCAAACCTCATGTCTGAGATGGCATAAATAGTCACACTCCTCCCCCCACCCCGTAACCAG
TCTTTGCACTTGGAAAGTTCACG[2]ACTGGCAGCCTGCAGTCTCCATGACTTAAGTAGGTTT
GCCACAGCTCATCTCTTCACCTCAGAAGACTCCAGACCTGGACTCACTCCATGCAGCAAA
GAAAGGTATGTTAAACATTGGGAAAAACTCAGATCATTTAAGTACCTAGATCTGTGAGCC
ATTCTCAAAGAGAAGGGGAGA**G**[3]AGGGGAAGGGACCCAAATCATACGCAAAACAATTTTTT
TTTTTTTTTTTAGAATCTAAAGAAGCGTTTTGGTAGTCTCAAATGTCACTTCTTTGAAGA
TAAAGTCAGACTTTTACAATAATGCTGGAAATGGTTTTCTGGAGTGTATCTCTTGTCTGT
ATAATTTTGAGGAAGTAAATACTATTAATAGGTGTTTTCAACAGCATATATTAAAGCAGC
CTTTCCAGCACGTTTTGAAGCGTCAATCCTTTGCTGTCTTTGCAAGGTAAAGTTTGGTGT
TTTCTCTTTATCTTAG**TTGATGCTACTGGGTCCATCTCAGGCTGATCTTGACACCTTCCA**
TGTTCTGCTCTCTTCAGCCAGACTTGTCTGTTTCATTTTGGAAGAAGACTAAAAATGGTA
AGGACAGCTAAGAGATCCCTAAAAAGTGTTATTTGTCCTTTTTTGTGGGAAGAACTAAAA
CTGACTAGCAAGAGAGATGTTTAAGTTATCTGTACTTCAGTTATTTACTGAAGAAAACTT

TCATGATCTTTCATAAACAAGATTAATCATGGGGGTATTGTGTAATTTCAATTAATTAAG
ATTAATCAATACTTGCTGTGTTCTCCTTCCTATGGACTTCCCTAGTGGCTCAGATAGTAG
TAATTTCAATTAAGA

LWLT01000021.1_50703293_T/C synonymous variant

**GTCCTAGATATAAGTAGTAACAGCCATTATTTTCAATCAGAAGGAATTACTCACATGCTA
AATTTTACCAAGAACCTCAAGGTTCTGAAGAAACTGATGATGAACTATAATGACATCGCT
ACCTCCACCAGCAGGACCATGGAGAGTGAATCTCTTCAAATCCTGGAGTTCAGAGGCAAC
CATTTGGATATTTTATGGAGAGATGGTGATAACAGATACTTAAAATTCTTCAAGAATCTG
CTAAACTTAGAAGAGCTAGACATCTCTGAAAATTCTCTGAGTTTCTTACCTTTGGGAGTT
TTTGACAGTATGCCTCCAAATCTAAAGACTCTCTCCTTGGCCAAAAATGGGCTCAAGTCT
TTCAGTTGGAAAGGACTACAGAGTCTGAAGAATCTAGAAACTTTGGACCTCAGCTTCAAC
CAGCTGAAGACTGTCCCTGAGAGATTATCCAACTGTTCCCGCAGCCTCAAGAAACTCATA
CTTAAGAATAATCAAATCAGGTGCCTGACAAAGTATTTTCTCCAAGGTGCTTTCCAGTTG
CGACATCTGGACCTCAGCTCAAATAAAATTCAGGTTATCCAAAAGACGAGTTTTCCAGAA
AACGTCCTCAACAATCTAAACATTTTGTTTTTGCATCACAATCGATTTCTGTGCAACTGT
GATGCTGTGTGGTTTGTCTGGTGGGTTAACCATACCGAGGTGACTATTCCTTACTTGGCC
ACAGATGTGACTTGCATGGGACCAGGAGCACACAAGGGCCAGAGTGTGGTCTCTCTGGAT
CTATATACCTGTGAGTTAGATTTGACTAACTTCATCCTGTTCTCACTTTCCATATCAGCA
ATTCTCTCTCTGATGATGATCACAACAGCAAACCATCTCTATTTCTGGGATGTGTGGTAT
AGTTATCATTTCTGTAAAGCCAAAATAAAAGGGTATCGACGTCTGATATCACCCAATTCT
TGCTATGATGCTTTCATTGTATATGACACTAAAGACCCAGCAGTGACAGAGTGGGTTTTG
GACGAGCTGGTGGCCAAATTGGAAGACCCGAGAGAGAAGTGTTTTAATTTATGTCTTGAG
GAAAGAGACTGGTTACCAGGGCAGCCTGTTCTGGAAAATCTTTCCCAGAGCATACAGCTT
AGCAAAAAGACAGTGTTTGTGATGACAGACAAGTACGCAAAGACTGAGAATTTTAAGATA
GCATTTTACTTATCCCATCAGAGGCTCATGGAT**

LWLT01000021.1_50702074_T/C 3′UTR variant

**CATTTTACTTATCCCATCAGAGGCTCATGGATGAAAAAGTGGATGTAATCATCTTGATA
TTCCTTGAGAAGCCCCTTCAGAAGTCCAAGTTTCTCCAACTCCGGAAGAGGCTCTGTGGC
AGTTCTGTCCTTGAGTGGCCAACAAACCCACAGGCTCACCCGTACTTCTGGCAGTGTCTG
AAAAATGCCCTGGCCACAGACAATCACGTGACCTACAGTCAGGTGTTCAAAGAGACAGCC
TAGCCCTTCTTTGCCGAATGTGACTGCTACCAAGGAGAAGCTCAGCTGCCTTGATCGGCT
CACCCATGCCTTGCCAGAAGAGTGTTCTAAGATTCTCCAAGCCCTGGGATTGCCCATATT
GGAGAGGAGTCACCAATACATAACAAAGGAAGCAGAAAAATGGGATTTATATAAGCATCA
AGTCATCTTTCTCAGCTCTCTCTGTCTCCATTTGCACTTGAGTCTTTGTCTTCTGCCCTT
GCATAAAATACTGTTGGGAGAAGGGTGGCAAGTAGAGGATGTGGGACTTTGATTCTCCTG
TAATTGTGATTATTACATATACACACAGTCACCAAGAACTGCACTTCTACCCTCAAGAGG
CACTGGTATGTATAGAAATAGGGTTAAAAAAAAGGCTCAGAGTCTGCTTATATGACATTA
AAATGTACCAGTTAATTAGTGGTAAAAATAAAGACACAGTTAACTCCTCAGCCAATTGAC
TCAACCATCTAGTGCCCCGTTCTGTGCAGACCAAGTGCTGGCCCCCAGCAGGTGGTACAG
CTCAAGTGCTCCTTGCTCTTTCTCTCCTGGGCCTGTTACTGGGTTTTTTGGGGAAATAGA
AACATCTTTGTCATCGATGGACATAGTCTACTTACAAGTGGGAGAAGAATAAAGTCATGC
TCTTTACTTTTGATGATAATTTACCTGCTTAAATATGGCTCTGGACTAAATGTTGAAATC
CTAATCCCCCAGTGTGATGGTGTTGGGAGGTGGGAGGTGGGGTTTTTAAGAAGTGGTTAG
GTCATGAGACTGGAGCCCTCGTGAT**

LWLT01000021.1_50701297_C/T 3′UTR variant

**CTCAAGTGCTCCTTGCTCTTTCTCTCCTGGGCCTGTTACTGGGTTTTTTGGGGAAATAGA
AACATCTTTGTCATCGATGGACATAGTCTACTTACAAGTGGGAGAAGAATAAAGTCATGC
TCTTTACTTTTGATGATAATTTACCTGCTTAAATATGGCTCTGGACTAAATGTTGAAATC
CTAATCCCCCAGTGTGATGGTGTTGGGAGGTGGGAGGTGGGGTTTTTAAGAAGTGGTTAG
GTCATGAGACTGGAGCCCTCGTGATGGGATTACTGCCTTAGAAGAAGCAGTCAGGGGACT
AACTTGCTGTGTTCCCATCGTGTGAGGGTATGGAGAGAGGCTGGCAGTCTATAACACACA
GGAGGACCCTCAGCAGAACCAGATCATATTGACACTCTGATCTCCTTCAGACTGGCTTCC
ATGCTTCAGACTTGTGAGAAATAAATGTTTGTTCTTTAAGTCATGCAGTTTGTGATGTTT**

TGTTACAGCAGCTGAGCTAAGACAGATTTTTTTAATCTGCATGGCAAAGTACTGTTTCCA
AAGAAAAGTTGCCTCATCCAATGATCTTTCAAACTGCTTTATTAACTAATACTGTATACT
TGTAATTATCTGAAAACTTGATCCATCAATGTCAGACAGCTTTGATGATAAACTCTAAAG
GAGAACCCAAGAGCATATATTTATTTAGAGTTAGCAAGCAGGGATGACAGTCACATGGAT
