# Supplementary material for: Single Nucleotide Polymorphisms in Genes Encoding Toll-Like Receptors 7 and 8 and Their Association with Proviral Load of SRLVs in Goats of Polish Carpathian Breed
Source: Animals (Basel). 2021 Jun 26;11(7):1908. doi: 10.3390/ani11071908 (PMC8300119; doi:10.3390/ani11071908)
Supplement: Supplementary file 1 [file animals-11-01908-s001.zip › Supplementary Figure S2. The exact TLR8 SNPs localization based on transcript and genomic sequence..pdf]

Gene: TLR8 ENSCHIG00000016625
Transcript: TLR8-201 ENSCHIT00000024133.1
Genome assembly: ARS1 (GCA_001704415.1) Ensembl

cDNA sequence with SNP localization:

```
  1 ATGACCCTTCGCTTTTTGCTTCTGACCTCCCTTTTCCTGCTCATCTCCGATTCCTGTGAG     60
  1 ATGACCCTTCGCTTTTTGCTTCTGACCTCCCTTTTCCTGCTCATCTCCGATTCCTGTGAG     60
  1 -M--T--L--R--F--L--L--L--T--S--L--F--L--L--I--S--D--S--C--E-     20

 61 TTCTTCACTGAAGCCAGTTATCCCCGAAGCTATCCTTGCGATGTGAAAAACAAAAATGGC    120
 61 TTCTTCACTGAAGCCAGTTATCCCCGAAGCTATCCTTGCGATGTGAAAAACAAAAATGGC    120
 21 -F--F--T--E--A--S--Y--P--R--S--Y--P--C--D--V--K--N--K--N--G-     40

121 TCTTTTATTGCAGAATGTAACAGTCGTCGATTACAGGAAGTACCCCAAACAGTGGACAAA    180
121 TCTTTTATTGCAGAATGTAACAGTCGTCGATTACAGGAAGTACCCCAAACAGTGGACAAA    180
 41 -S--F--I--A--E--C--N--S--R--R--L--Q--E--V--P--Q--T--V--D--K-     60

181 GCTGTGACAGAAGTAGACCTGTCTGATAATTTCATCACACGTATAACGAATGAATCCTTT    240
181 GCTGTGACAGAAGTAGACCTGTCTGATAATTTCATCACACGTATAACGAATGAATCCTTT    240
 61 -A--V--T--E--V--D--L--S--D--N--F--I--T--R--I--T--N--E--S--F-     80

241 CAAGGGCTGCAAAATCTGACTAAAATCAACCTGAACCATAATGCCAAGTCCCAGAGTGGA    300
241 CAAGGGCTGCAAAATCTGACTAAAATCAACCTGAACCATAATGCCAAGTCCCAGAGTGGA    300
 81 -Q--G--L--Q--N--L--T--K--I--N--L--N--H--N--A--K--S--Q--S--G-    100

301 AATCCTGCTGTAAAGAAAGCTATGACTATTACAGACGGGGCATTTCTCAACCTCAAACAC    360
301 AATCCTGCTGTAAAGAAAGCTATGACTATTACAGACGGGGCATTTCTCAACCTCAAACAC    360
101 -N--P--A--V--K--K--A--M--T--I--T--D--G--A--F--L--N--L--K--H-    120

361 CTAAGGGAGTTGCTGCTGGAAGACAACCAGTTACAAGAAATACCGGCTGGTTTGCCAGAA    420
361 CTAAGGGAGTTGCTGCTGGAAGACAACCAGTTACAAGAAATACCGGCTGGTTTGCCAGAA    420
121 -L--R--E--L--L--L--E--D--N--Q--L--Q--E--I--P--A--G--L--P--E-    140

421 TCTTTGAAAGAACTTAGTCTAATTCAAAACAATATAATTACGTTAACGAAAAAGAACACT    480
421 TCTTTGAAAGAACTTAGTCTAATTCAAAACAATATAATTACGTTAACGAAAAAGAACACT    480
141 -S--L--K--E--L--S--L--I--Q--N--N--I--I--T--L--T--K--K--N--T-    160

                   LWLT01000021.1_50666071_A/G synonymous variant

481 TCTGGACTTGGGAACCTGGAACGTCTCTATTTGGGCTGGAACTGTTATTTTGCTTGTAGT    540
481 TCTGGACTTGGGAACCTGGAACGTCTCTATTTGGGCTGGAACTGTTATTTTGCTTGTAGT    540
161 -S--G--L--G--N--L--E--R--L--Y--L--G--W--N--C--Y--F--A--C--S-    180

541 AAAAAATTTAGCATAGAAAATGGAGCATTCCAAAACCTTACCAAGTTGAAGGTGCTGTCA    600
541 AAAAAATTTAGCATAGAAAATGGAGCATTCCAAAACCTTACCAAGTTGAAGGTGCTGTCA    600
181 -K--K--F--S--I--E--N--G--A--F--Q--N--L--T--K--L--K--V--L--S-    200

601 TTATCTTTTAATCCCCTTCACAGCGTGCCACCGAGTCTGCCAAGCTCGCTAACAGAACTC    660
```

```
601  TTATCTTTTAATCCCCTTCACAGCGTGCCACCGAGTCTGCCAAGCTCGCTAACAGAACTC    660
201  -L--S--F--N--P--L--H--S--V--P--P--S--L--P--S--S--L--T--E--L-    220

661  TACCTTAGCAATACCCATATTGGAAACGTCAGTGAAGAAGACTTCAAGGAACTGAGCAAT    720
661  TACCTTAGCAATACCCATATTGGAAACGTCAGTGAAGAAGACTTCAAGGAACTGAGCAAT    720
221  -Y--L--S--N--T--H--I--G--N--V--S--E--E--D--F--K--E--L--S--N-    240

721  TTAAGAGTACTAGATTTAAGTGGAAACTGCCCGAGATGTTTTAACGCTCCGTTTCCCTGT    780
721  TTAAGAGTACTAGATTTAAGTGGAAACTGCCCGAGATGTTTTAACGCTCCGTTTCCCTGT    780
241  -L--R--V--L--D--L--S--G--N--C--P--R--C--F--N--A--P--F--P--C-    260

781  GTACCTTGCCAAGGAGGTGCTTCAATTCAGATACACCCTCTTGCTTTTCAAACTCTGACC    840
781  GTACCTTGCCAAGGAGGTGCTTCAATTCAGATACACCCTCTTGCTTTTCAAACTCTGACC    840
261  -V--P--C--Q--G--G--A--S--I--Q--I--H--P--L--A--F--Q--T--L--T-    280

841  CAACTTCGCTACCTAAACCTCTCTAGCACTTCGCTCCGGAAGGTTCCTGCCAGCTGGTTT    900
841  CAACTTCGCTACCTAAACCTCTCTAGCACTTCGCTCCGGAAGGTTCCTGCCAGCTGGTTT    900
281  -Q--L--R--Y--L--N--L--S--S--T--S--L--R--K--V--P--A--S--W--F-    300

901  GACAACATGCACAATCTGAAGGTGTTGGATCTTGAATTCAACTATTTAATGGACGAAATA    960
901  GACAACATGCACAATCTGAAGGTGTTGGATCTTGAATTCAACTATTTAATGGACGAAATA    960
301  -D--N--M--H--N--L--K--V--L--D--L--E--F--N--Y--L--M--D--E--I-    320

961  GCCTCGGGGGAATTTTTGACAAAATTGCCCTCCTTAGAAATACTTGATTTATCTTACAAC   1020
961  GCCTCGGGGGAATTTTTGACAAAATTGCCCTCCTTAGAAATACTTGATTTATCTTACAAC   1020
321  -A--S--G--E--F--L--T--K--L--P--S--L--E--I--L--D--L--S--Y--N-    340

1021 TACGAACTGAAAAAATATCCTCAGCACATTAACATTTCCAAAAATTTCTCAAAGCTTATA   1080
1021 TACGAACTGAAAAAATATCCTCAGCACATTAACATTTCCAAAAATTTCTCAAAGCTTATA   1080
341  -Y--E--L--K--K--Y--P--Q--H--I--N--I--S--K--N--F--S--K--L--I-    360

1081 TCTCTCCAGATGTTGCATTTAAGAGGTTATGTGTTCCAGGAACTTAGAAGGAAAGATTTC   1140
1081 TCTCTCCAGATGTTGCATTTAAGAGGTTATGTGTTCCAGGAACTTAGAAGGAAAGATTTC   1140
361  -S--L--Q--M--L--H--L--R--G--Y--V--F--Q--E--L--R--R--K--D--F-    380

1141 AAGCCCCTGCAGCACCTCTCAAATTTAACGACTATCAACTTGGGTGTTAACTTTATTAAG   1200
1141 AAGCCCCTGCAGCACCTCTCAAATTTAACGACTATCAACTTGGGTGTTAACTTTATTAAG   1200
381  -K--P--L--Q--H--L--S--N--L--T--T--I--N--L--G--V--N--F--I--K-    400

1201 CAGATTGATTTTAGCATTTTCCACTGGTTCCCCAACCTGAAAATCGTTTACTTGTCAGAA   1260
1201 CAGATTGATTTTAGCATTTTCCACTGGTTCCCCAACCTGAAAATCGTTTACTTGTCAGAA   1260
401  -Q--I--D--F--S--I--F--H--W--F--P--N--L--K--I--V--Y--L--S--E-    420

1261 AACAGAATATCGCCCTTGGTCAGTGATACTGAGCAACATGATGCAAATAGGACCTTTTTC   1320
1261 AACAGAATATCGCCCTTGGTCAGTGATACTGAGCAACATGATGCAAATAGGACCTTTTTC   1320
421  -N--R--I--S--P--L--V--S--D--T--E--Q--H--D--A--N--R--T--F--F-    440

1321 CAGAGTCACATCCTGAAGCGACGCTCAGCTGATATTCAATTTGACCCACATTCGAATTTT   1380
1321 CAGAGTCACATCCTGAAGCGACGCTCAGCTGATATTCAATTTGACCCACATTCGAATTTT   1380
441  -Q--S--H--I--L--K--R--R--S--A--D--I--Q--F--D--P--H--S--N--F-    460
```

```
1381 TATCATAACACCCATCCTTTAATAAAGACAGAATGTTCACGTCTTGGCAATGCCTTAGAT  1440
1381 TATCATAACACCCATCCTTTAATAAAGACAGAATGTTCACGTCTTGGCAATGCCTTAGAT  1440
 461 -Y--H--N--T--H--P--L--I--K--T--E--C--S--R--L--G--N--A--L--D-   480

1441 TTAAGCTTGAACAGTATTTTCTTTATTGGGGTAAACCAGTTTAAAGATTTTGGAAACATT  1500
1441 TTAAGCTTGAACAGTATTTTCTTTATTGGGGTAAACCAGTTTAAAGATTTTGGAAACATT  1500
 481 -L--S--L--N--S--I--F--F--I--G--V--N--Q--F--K--D--F--G--N--I-   500

1501 TCCTGTTTAAATCTGTCTTCAAATGGCAATGGCCAGGTGTTAAATGGAACGGAATTTTCA  1560
1501 TCCTGTTTAAATCTGTCTTCAAATGGCAATGGCCAGGTGTTAAATGGAACGGAATTTTCA  1560
 501 -S--C--L--N--L--S--S--N--G--N--G--Q--V--L--N--G--T--E--F--S-   520

1561 CGCTTGTCTGGTATCAAGTATTTGGATTTGACAAACAATAGACTAGACTTTGATGATGAT  1620
1561 CGCTTGTCTGGTATCAAGTATTTGGATTTGACAAACAATAGACTAGACTTTGATGATGAT  1620
 521 -R--L--S--G--I--K--Y--L--D--L--T--N--N--R--L--D--F--D--D--D-   540

1621 GCTGCTTTCAGTGAGTTGCCATTGTTGGAAGTTCTCGATCTCAGCTACAATGCGCACTAT  1680
1621 GCTGCTTTCAGTGAGTTGCCATTGTTGGAAGTTCTCGATCTCAGCTACAATGCGCACTAT  1680
 541 -A--A--F--S--E--L--P--L--L--E--V--L--D--L--S--Y--N--A--H--Y-   560

1681 TTCCGAATAGCAGGGGTAACGCACCGTCTAGGATTTATTGAGCATTTAACTAACCTGAGA  1740
1681 TTCCGAATAGCAGGGGTAACGCACCGTCTAGGATTTATTGAGCATTTAACTAACCTGAGA  1740
 561 -F--R--I--A--G--V--T--H--R--L--G--F--I--E--H--L--T--N--L--R-   580

1741 GTTTTAAACTTGAGCAACAATGACATTTTTACTTTAACAGAAACACAACTGAAAAGCGCG  1800
1741 GTTTTAAACTTGAGCAACAATGACATTTTTACTTTAACAGAAACACAACTGAAAAGCGCG  1800
 581 -V--L--N--L--S--N--N--D--I--F--T--L--T--E--T--Q--L--K--S--A-   600

1801 TCCCTAGGAGAATTAGTTTTCAGTGGGAACCGCCTTGACCTTCTGTGGAATGCTCAAGAT  1860
1801 TCCCTAGGAGAATTAGTTTTCAGTGGGAACCGCCTTGACCTTCTGTGGAATGCTCAAGAT  1860
 601 -S--L--G--E--L--V--F--S--G--N--R--L--D--L--L--W--N--A--Q--D-   620
```

LWLT01000021.1_50664682_A/G synonymous variant

```
1861 GTCAGGTACTGGCAAATTTTTCAAAATCTCACCAATCTGACCCGGCTTGACTTAGCCCGT  1920
1861 GTCAGGTACTGGCAAATTTTTCAAAATCTCACCAATCTGACCCGGCTTGACTTAGCCCGT  1920
 621 -V--R--Y--W--Q--I--F--Q--N--L--T--N--L--T--R--L--D--L--A--R-   640

1921 AATAACCTTCAGCATATCTCCAGTCAGACCTTCCTTAACTTGCCCAGGACTCTCACTGAC  1980
1921 AATAACCTTCAGCATATCTCCAGTCAGACCTTCCTTAACTTGCCCAGGACTCTCACTGAC  1980
 641 -N--N--L--Q--H--I--S--S--Q--T--F--L--N--L--P--R--T--L--T--D-   660

1981 CTATATATAAATGATAACATGTTAAATTTCTTTAACTGGTCATTACTGGAATACTTCCCT  2040
1981 CTATATATAAATGATAACATGTTAAATTTCTTTAACTGGTCATTACTGGAATACTTCCCT  2040
 661 -L--Y--I--N--D--N--M--L--N--F--F--N--W--S--L--L--E--Y--F--P-   680

2041 CACCTCAGGTTGCTTGACTTAAGTGGAAACCAGCTGTTCTTTTTAACCAATAGCCTATCT  2100
2041 CACCTCAGGTTGCTTGACTTAAGTGGAAACCAGCTGTTCTTTTTAACCAATAGCCTATCT  2100
 681 -H--L--R--L--L--D--L--S--G--N--Q--L--F--F--L--T--N--S--L--S-   700
```

```
2101 ACATTTGCATCTTCTCTTGAGACGCTACTACTGAGTCGAAACAGAATTTCCCACCTGCCA   2160
2101 ACATTTGCATCTTCTCTTGAGACGCTACTACTGAGTCGAAACAGAATTTCCCACCTGCCA   2160
 701 -T--F--A--S--S--L--E--T--L--L--L--S--R--N--R--I--S--H--L--P-    720

2161 TCTGACTTTCTTTCTGGAGCCAGCAGCCTGATACACCTCGATTTGAGCTCCAACCAGCTC   2220
2161 TCTGACTTTCTTTCTGGAGCCAGCAGCCTGATACACCTCGATTTGAGCTCCAACCAGCTC   2220
 721 -S--D--F--L--S--G--A--S--S--L--I--H--L--D--L--S--S--N--Q--L-    740

2221 AAGATGCTCAACAGATCCACGTTTGAAACGAAGACCGCCACCAAGTTAACCATTTTGGAA   2280
2221 AAGATGCTCAACAGATCCACGTTTGAAACGAAGACCGCCACCAAGTTAACCATTTTGGAA   2280
 741 -K--M--L--N--R--S--T--F--E--T--K--T--A--T--K--L--T--I--L--E-    760
```

LWLT01000021.1_50664208_C/T synonymous variant

```
2281 CTAGGGAGTAACCCTTTTGACTGTACCTGTGACTTCGGAGATTTTCGAGAATGGATGGAC   2340
2281 CTAGGGAGTAACCCTTTTGACTGTACCTGTGACTTCGGAGATTTTCGAGAATGGATGGAC   2340
 761 -L--G--S--N--P--F--D--C--T--C--D--F--G--D--F--R--E--W--M--D-    780

2341 GGAAATCTGAACGTCAGGGTTCCCAGACTGACCGATGTCATTTGTGCTAGTCCTGGGGAT   2400
2341 GGAAATCTGAACGTCAGGGTTCCCAGACTGACCGATGTCATTTGTGCTAGTCCTGGGGAT   2400
 781 -G--N--L--N--V--R--V--P--R--L--T--D--V--I--C--A--S--P--G--D-    800

2401 CAAGAAGGCAAGAGCATTGTGAGTCTAGACCTCACCACTTGTGTTTCAGATACCATTGCG   2460
2401 CAAGAAGGCAAGAGCATTGTGAGTCTAGACCTCACCACTTGTGTTTCAGATACCATTGCG   2460
 801 -Q--E--G--K--S--I--V--S--L--D--L--T--T--C--V--S--D--T--I--A-    820
```

LWLT01000021.1_50664064_C/T synonymous variant

```
2461 GCCATATTCTGTTTCTTAACCTTTTCTGTCACCATCTCAGTGATGCTGGCTGCCCTGGCC   2520
2461 GCCATATTCTGTTTCTTAACCTTTTCTGTCACCATCTCAGTGATGCTGGCTGCCCTGGCC   2520
 821 -A--I--F--C--F--L--T--F--S--V--T--I--S--V--M--L--A--A--L--A-    840

2521 CACCACTGGTTTTACTGGGATGCTTGGTTTATCTACCATGTGTGCTTAGCTAAGGTCAAA   2580
2521 CACCACTGGTTTTACTGGGATGCTTGGTTTATCTACCATGTGTGCTTAGCTAAGGTCAAA   2580
 841 -H--H--W--F--Y--W--D--A--W--F--I--Y--H--V--C--L--A--K--V--K-    860

2581 GGCTACAGGTCTCTGTCCACATCCCAGACTTTCTACGATGCTTATGTTTCTTATGACACC   2640
2581 GGCTACAGGTCTCTGTCCACATCCCAGACTTTCTACGATGCTTATGTTTCTTATGACACC   2640
 861 -G--Y--R--S--L--S--T--S--Q--T--F--Y--D--A--Y--V--S--Y--D--T-    880

2641 AAAGACGCTTCTGTCACGGACTGGGTGATCAATGAATTGCGCTTCCACCTGGAAGAGAGT   2700
2641 AAAGACGCTTCTGTCACGGACTGGGTGATCAATGAATTGCGCTTCCACCTGGAAGAGAGT   2700
 881 -K--D--A--S--V--T--D--W--V--I--N--E--L--R--F--H--L--E--E--S-    900

2701 GAGGACAAGAACGTGCTCCTGTGTTTAGAGGAAAGGGATTGGGACCCGGGTCTAGCCATC   2760
2701 GAGGACAAGAACGTGCTCCTGTGTTTAGAGGAAAGGGATTGGGACCCGGGTCTAGCCATC   2760
 901 -E--D--K--N--V--L--L--C--L--E--E--R--D--W--D--P--G--L--A--I-    920

2761 ATCGACAACCTCATGCAGAGCATCAACCAAAGCAAGAAAACAATATTTGTTTTAACCAAA   2820
```

```
2761 ATCGACAACCTCATGCAGAGCATCAACCAAAGCAAGAAAACAATATTTGTTTTAACCAAA   2820
 921 -I--D--N--L--M--Q--S--I--N--Q--S--K--K--T--I--F--V--L--T--K-   940

2821 AAATATGCCAAAAACTGGAATTTTAAAACGGCATTCTACTTGGCCTTGCAGAGGCTAATG   2880
2821 AAATATGCCAAAAACTGGAATTTTAAAACGGCATTCTACTTGGCCTTGCAGAGGCTAATG   2880
 941 -K--Y--A--K--N--W--N--F--K--T--A--F--Y--L--A--L--Q--R--L--M-   960

2881 GAGGAGAATATGGATGTGATTGTCTTTATTCTGCTGGAGCCAGTGTTGCAGCATTCGCAG   2940
2881 GAGGAGAATATGGATGTGATTGTCTTTATTCTGCTGGAGCCAGTGTTGCAGCATTCGCAG   2940
 961 -E--E--N--M--D--V--I--V--F--I--L--L--E--P--V--L--Q--H--S--Q-   980

2941 TATTTGAGGCTGCGGCAGAGGATCTGCAAGAGTTCCATCCTCCAGTGGCCTGATAACCCC   3000
2941 TATTTGAGGCTGCGGCAGAGGATCTGCAAGAGTTCCATCCTCCAGTGGCCTGATAACCCC   3000
 981 -Y--L--R--L--R--Q--R--I--C--K--S--S--I--L--Q--W--P--D--N--P-   1000

3001 AAGGCGGAAGGCTTGTTTTGGCAGAGTCTGAAAAATGTCGTCCTAACAGCCAACGATTCA   3060
3001 AAGGCGGAAGGCTTGTTTTGGCAGAGTCTGAAAAATGTCGTCCTAACAGCCAACGATTCA   3060
1001 -K--A--E--G--L--F--W--Q--S--L--K--N--V--V--L--T--A--N--D--S-   1020

3061 CGGATCTTCTGTCCGTTGCAGTATGAATCTTGAAGACGAACCTTATGAAATTGACCTGAA   3120
3061 CGGATCTTCTGTCCGTTGCAGTATGAATCTTGA...........................   3093
1021 -R--I--F--C--P--L--Q--Y--E--S--*-...........................   1030

3121 AATAACTGGAAATCATTAGAGCATAAGAAAATAAAATCCCTTTATCTCATCTATCTTCTT   3180
     ............................................................
     ............................................................

3181 CAATTCACAATTTCTACTTAATGGACAGATGCTTTTTGAACAACCAATATGTATTGAGTC   3240
     ............................................................
     ............................................................

3241 CTAAGGTGGGTTCTGAAATGCCCAATATCTATCAGCTGTTGAGTGGATAAATAAAATGGG   3300
     ............................................................
     ............................................................

3301 CTATCACCATACACTGGAGTATTATTCAGCCTTAGAAAGGGATGGAGTATGACACACAGG   3360
     ............................................................
     ............................................................

3361 ACAACATGGATGTGCCTTGGAAACCAGATGCTCAGTGAAAGACGCCAGTCACAAGAAACC   3420
     ............................................................
     ............................................................

3421 ACATGTTGTCTGATGCCATATATAGGAAATGTCCAGAATAGGCAAATCCATAGAAACAGA   3480
     ............................................................
     ............................................................

3481 AACTGGGGAAGTGGTTCCCCCGGGGCTGGGGGAGCCAAGAGAAGATGGGAAGTGACTGCA   3540
     ............................................................
     ............................................................
```

3541 TATGGTCACAGGATGTCTTGTTGGAGTGAGGCCACATTCTCAAATTCATGATAGTGATGG 3600
....
....

3601 CTGCATAACTTTGTGAATCTCCTGACAGCCTTATTTCTCTTCTTGGCCATTTTTTCAGTG 3660
....
....

3661 GCTTTTCCAGCATGGCCTCACCATGTCTGAAGCTCCAGCATTTCTGTTCTACTCCTGAAG 3720
....
....

3721 TTGTCTCTATCAGCTCAGTTTTGTCTTCCTCTTTTGCGGGGGAAGCTGGTCAAGATTCGC 3780
....
....

3781 CAAATCCCTGTTTGGCACTGATCTCTGGTGGTTGGCCTCTGTAAAGAACCCTGAACCACA 3840
....
....

3841 GGAATTAGAATGCCCAGGTCTGATCATAGCAAATCACTCTGCGTTTCTTTTTTTTTCCTTT 3900
....
....

3901 TTCAAAAAATTTATTTGATTGTACTGGTCTTAGTTGTGACATGTGGGATCTAGTTCCCTG 3960
....
....

3961 ACTCCCTGGTCAAACCCGGGCCCCCTGCATTGGGAGCACCGAGTCTTAACCACTGGACCC 4020
....
....

4021 CCAGGGAAGTTCCCACTCCACCTTTCTGAGCATGATTCCTCATGTCTAGAATGAACTATG 4080
....
....

4081 TGATCTCTATGTGATTTTCCAACATTACAACTGCATTCGTTTCCTAGCATCACCAAAACA 4140
....
....

4141 AAGTGCCCCAAACTGGGTGGCTTAAAACAATCAAAGTTCATTGTCTCACAGTTCTGAGGG 4200
....
....

4201 CTACAAGTCTGAAATCTGAGTGTCTACGGGGCCATGGTTCCTCTGAAACTTGTAGCCGAG 4260
....
....

4261 GGTCCTTCCTTGCCTTTCCCAGCTCCTGATGTTGCTGGGAGTCCTCGGAGTCCCTTGGCC 4320
....
....

4321 TGTCGCTGCGTCACTCTGGTCTCTGCCTCC**G**CTGTTGCATGGCCACCTTCCCTCCCTGTC 4380
....
....

LWLT01000021.1_50659559_C/T 3'UTR variant

4381 TCTCTGCTCTTATAAGGATGTTAGTCCTGTTGGAGTCAGGGTCCACCCTCCTCCAACATG 4440
....
....

4441 GCTGTGTTTTAACCAATAACATCTGCAGCAGCTCTGTTTCCAAATGAGGTCATAGTCTTC 4500
....
....

4501 TTTTTTTGAAGGAAGTAGTGTTTTATTTCTTTTAATATTTATTTTTCTTATTTATTTGAC 4560
....
....

4561 TGC**G**CTCTTAGTTGCGGCATGTGGGATCTAGTTCTCTGACCAAGGATCAAACCCCAGCCC 4620
....
....

LWLT01000021.1_50659346_C/A 3'UTR variant

4621 CCTGCCTTGGGAACGTGGGGTCTTAACCCCTGGACCACCAGGGAAGTCCCTCCAAATGAG 4680
....
....

4681 GTCATACTCTGAGGTACTGGAAGTTAC**A**ACTTCAACATAAAATTTGGGAAGGACACAGTT 4740
....
....

LWLT01000021.1_50659202_T/C 3'UTR variant

4741 CAACTCCTAACAACAACTGTGGCTTTGCTTCTT**T**AGAAATTACTCAGAACTCCGTGAAAG 4800
....
....

LWLT01000021.1_50659136_A/C 3'UTR variant

4801 TTGAAAATGCCCTTGTGAAAGGGAAAGTCATTTGAGTATTGGAAGAATAAAGGCAATTCC 4860
....
....

4861 CCTCTCAGCTATTAATAAAGCTATAATAGTGATATGGCCAGGTTTCACCCTCATTATCCT 4920
....
....

4921 TGAACTTAAAGCCATAATGGTATCTATTATTTCCCATGAGCACAAAAGAAGGTCGTGCAG 4980
....
....

4981 TCGTGTTCTCTTTCTGCCCTTGAATGGGAGAATTGGTCAATCCATGTCTCACTCAGCAAT 5040
....
....

5041 CACGGTGCAGTCTTGGAGGAACAAGGTGAGGGAAAACTGGGTTTTGAACCAGAAGGGGCC 5100
....
....

5101 ATCACGGGACATCCTCTGCCAATGACCCCAAAGAGAAAATAGTCTTTATAAATGTCCCTT 5160
....
....

5161 CAGTGTGTGAAGAGATGAGAGGTACAAAGAGAAAAACAAAAACCCCACTTATGATAGCAG 5220
....
....

5221 AATATTGCCATGGGGAAGCTCTTTGGTTTTTTTTGAGCCTTTTCATTTCTAGTGAGTTGC 5280
....
....

5281 ATTTCTAGGAGAGAAAAGAAGACCCCTTTGTTTTCCCAGTCTTCCTCTCCTTTGACATTT 5340
....
....

5341 TACCTGGCCTATTTTAAGTATAACTCTCTATCTCAGTAATGATGATACCAGAAACTCAGA 5400
....
....

5401 GCTGATCAAGAGTGATCGATGTCTGGATTTGTAGAAGCTCAGAGGCAGTTCCCCAAGCCA 5460
....
....

5461 GACACCTGAGGCTCACTGTTGACTACTGCATCTTACTCCCATACCCAACCATTCATCTAG 5520
....
....

5521 CAGATTTCCGCCCCCCTCCTTGGCTGTGCCATGTCTTAGGTATGGCACGTGGGATCTAGC 5580
....
....

5581 CCTCTGAGCATGGATCGAACCCAGGGCCCCTGCATTGGGAGCAAAGTCTTAACCACTGGA 5640
....
....

5641 CCACCAGGGAAGTCCCCATCTAGCAGTCTTGACCTCTTAAATTTCTCTCCCATCCATCCT 5700
....
....

5701 CCGTCTCTGTCTCTATCATCACCATGCCTTATCCAAGCTACGAGCATCTCTTATTGGCTT 5760
....
....

5761 GCTGGTATCTACTCTGATCCCTTCCCCAGTTCAGTTCCAGCAGCCAGTGATTCGTGTAAA 5820
....
....

5821 AGATGCAACTACTGAATTAAATTTAAAATCTTAATCCTAAACAAGGCACACAGGACTCTC 5880
....
....

5881 TGTAACCTCCCATTGGCCTGCCCCTCAGGCCTCCTCTCTGTGCATCCCTGTCACTAGCTT 5940
....
....

5941 TTGGTTCATGCTGAGAACACACCGAACCCTCTTGCCTCAGTACCTCCACACAGGCTACTA 6000
....
....

6001 CCCTGAGGCCCCTCCTCCTCCAGGCTTCAATTTCCTAACTCCTGCTCATCCTACAGAGAG 6060
....
....

6061 AGGTTCACTTAGAGAGGCTGTTCCCTAACCTCCAAATCTCACCTAGACTATCATCTTAGC 6120
....
....

6121 ATTGATCACAAGTTTTATTGTGAAGACCACAGAGAAATGGATAAAGTGCTTTTAACGCAT 6180
....
....

6181 GGGTCTTCCATTAGACTGTGGCCTCTAGAGATGAGAGAAGGTTCCTTTCGGTCCATCATT 6240
....
....

6241 GTGTCCCC

Genomic DNA (reverse strand) sequence with SNP and primers localization:

LWLT01000021.1_50666071_A/G synonymous variant

CCTGAACCATAATGCCAAGTCCCAGAGTGGAAATCCTGCTGTAAAGAAAGCTATGACTAT
TACAGACGGGGCATTTCTCAACCTCAAACACCTAAGGGAGTTGCTGCTGGAAGACAACCA
GTTACAAGAAATACCGGCTGGTTTGCCAGAATCTTTGAAAGAACTTAGTCTAATTCAAAA
CAATATAATTACGTTAACGAAAAAGAACACTTCTGGACTTGGGAACCTGGAACGTCTCTA
TTTGGGCTGGAACTGTTATTTTGCTTGTAGTAAAAAATTTAGCATAGAAAATGGAGCATT
CCAAAACCTTACCAAGTTGAAGGTGCTGTCATTATCTTTTAATCCCCTTCACAGCGTGCC
ACCGAGTCTGCCAAGCTCGCTAACAGAACTCTACCTTAGCAATACCCATATTGGAAACGT
CAGTGAAGAAGACTTCAAGGAACTGAGCAATTTAAGAGTACTAGATTTAAGTGGAAACTG

LWLT01000021.1_50664682_A/G synonymous variant

AGTTCTCGATCTCAGCTACAATGCGCACTATTTCCGAATAGCAGGGGTAACGCACCGTCT
AGGATTTATTGAGCATTTAACTAACCTGAGAGTTTTAAACTTGAGCAACAATGACATTTT
TACTTTAACAGAAACACAACTGAAAAGCGCGTCCCTAGGAGAATTAGTTTTCAGTGGGAA
CCGCCTTGACCTTCTGTGGAATGCTCAAGATGTCAGGTACTGGCAAATTTTTCAAAATCT
CACCAATCTGACCCGGCTTGACTTAGCCCGTAATAACCTTCAGCATATCTCCAGTCAGAC
CTTCCTTAACTTGCCCAGGACTCTCACTGACCTATATATAAATGATAACATGTTAAATTT
CTTTAACTGGTCATTACTGGAATACTTCCCTCACCTCAGGTTGCTTGACTTAAGTGGAAA
CCAGCTGTTCTTTTTAACCAATAGCCTATCTACATTTGCATCTTCTCTTGAGACGCTACT

[1]LWLT01000021.1_50664283_C/T synonymous variant

[2]LWLT01000021.1_50664064_C/T synonymous variant

CTTTAACTGGTCATTACTGGAATACTTCCCTCACCTCAGGTTGCTTGACTTAAGTGGAAA
CCAGCTGTTCTTTTTAACCAATAGCCTATCTACATTTGCATCTTCTCTTGAGACGCTACT
ACTGAGTCGAAACAGAATTTCCCACCTGCCATCTGACTTTCTTTCTGGAGCCAGCAGCCT
GATACACCTCGATTTGAGCTCCAACCAGCTCAAGATGCTCAACAGATCCACGT[1]TTGAAAC
GAAGACCGCCACCAAGTTAACCATTTTGGAACTAGGGAGTAACCCTTTTGACTGTACCTG
TGACTTCGGAGATTTTCGAGAATGGATGGACGGAAATCTGAACGTCAGGGTTCCCAGACT
GACCGATGTCATTTGTGCTAGTCCTGGGGATCAAGAAGGCAAGAGCATTGTGAGTCTAGA
CCTCACCACTTGTGTTTCAGATACCATTGCG[2]GCCATATTCTGTTTCTTAACCTTTTCTGT
CACCATCTCAGTGATGCTGGCTGCCCTGGCCCACCACTGGTTTTACTGGATGCTTGGTT
TATCTACCATGTGTGCTTAGCTAAGGTCAAAGGCTACAGGTCTCTGTCCACATCCCAGAC
TTTCTACGATGCTTATGTTTCTTATGACACCAAAGACGCTTCTGTCACGGACTGGGTGAT

[1]LWLT01000021.1_50659559_C/T 3’UTR variant

[2]LWLT01000021.1_50659346_C/A 3’UTR variant

[3]LWLT01000021.1_50659202_T/C 3’UTR variant

[4]LWLT01000021.1_50659136_A/C 3’UTR variant

GAGGGCTACAAGTCTGAAATCTGAGTGTCTACGGGGCCATGGTTCCTCTGAAACTTGTAG
CCGAGGGTCCTTCCTTGCCTTTCCCAGCTCCTGATGTTGCTGGGAGTCCTCGGAGTCCCT
TGGCCTGTCGCTGCGTCACTCTGGTCTCTGCCTCCG[1]CTGTTGCATGGCCACCTTCCCTCC
CTGTCTCTCTGCTCTTATAAGGATGTTAGTCCTGTTGGAGTCAGGGTCCACCCTCCTCCA
ACATGGCTGTGTTTTAACCAATAACATCTGCAGCAGCTCTGTTTCCAAATGAGGTCATAG
TCTTCTTTTTTTGAAGGAAGTAGTGTTTTATTTCTTTTAATATTTATTTTTCTTATTTAT
TTGACTGCG[2]CTCTTAGTTGCGGCATGTGGGATCTAGTTCTCTGACCAAGGATCAAACCCC
AGCCCCCTGCCTTGGGAACGTGGGGTCTTAACCCCTGGACCACCAGGGAAGTCCCTCCAA
ATGAGGTCATACTCTGAGGTACTGGAAGTTACA[3]ACTTCAACATAAAATTTGGGAAGGACA
CAGTTCAACTCCTAACAACAACTGTGGCTTTGCTTCTTT[4]AGAAATTACTCAGAACTCCGT
GAAAGTTGAAAATGCCCTTGTGAAAGGGAAAGTCATTTGAGTATTGGAAGAATAAAGGCA
ATTCCCCTCTCAGCTATTAATAAAGCTATAATAGTGATATGGCCAGGTTTCACCCTCATT
