# Supplementary material for: Single Nucleotide Polymorphisms in Genes Encoding Toll-Like Receptors 7 and 8 and Their Association with Proviral Load of SRLVs in Goats of Polish Carpathian Breed
Source: Animals (Basel). 2021 Jun 26;11(7):1908. doi: 10.3390/ani11071908 (PMC8300119; doi:10.3390/ani11071908)
Supplement: Supplementary file 1 [file animals-11-01908-s001.zip › Supplementary Figure S3.pdf]

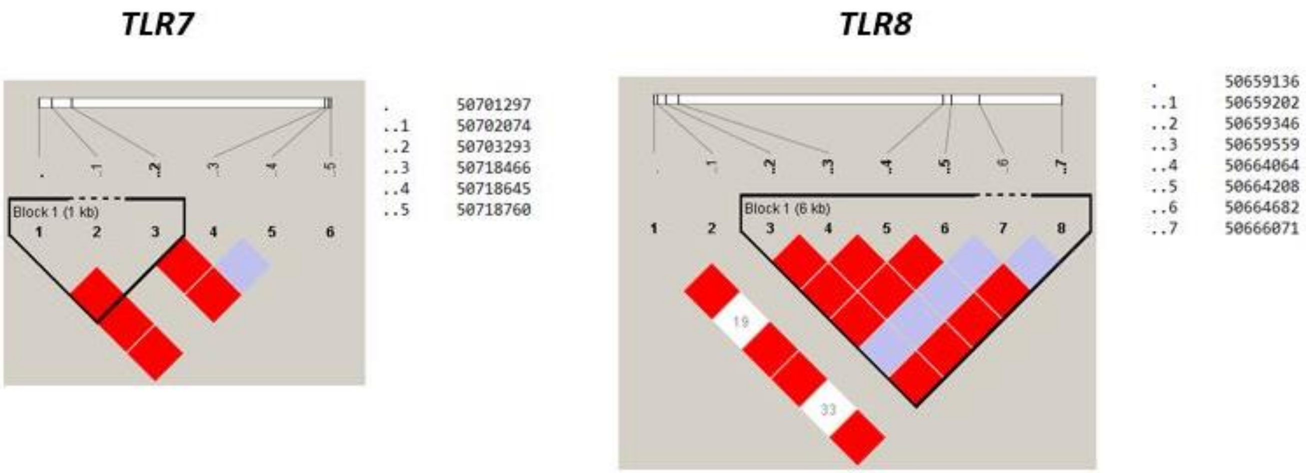

Figure S3. The visualization of LD and haplotype blocks based on Haploview software for TLR7 and TLR8 genes.
