# Supplementary material for: Single Nucleotide Polymorphisms in Genes Encoding Toll-Like Receptors 7 and 8 and Their Association with Proviral Load of SRLVs in Goats of Polish Carpathian Breed
Source: Animals (Basel). 2021 Jun 26;11(7):1908. doi: 10.3390/ani11071908 (PMC8300119; doi:10.3390/ani11071908)
Supplement: Supplementary file 1 [file animals-11-01908-s001.zip › Supplementary Table S1-updated.pdf]

Supplementary Table S1. The linkage disequilibrium (LD) between identified SNPs in TLRs genes.

**SNP1**	**SNP2**	**r^2**
TLR7		
LWLT01000021.1-50701297	LWLT01000021.1-50702074	0.854
LWLT01000021.1-50701297	LWLT01000021.1-50703293	**0.173**
LWLT01000021.1-50701297	LWLT01000021.1-50718645	0.731
LWLT01000021.1-50702074	LWLT01000021.1-50703293	**0.148**
LWLT01000021.1-50702074	LWLT01000021.1-50718645	0.856
LWLT01000021.1-50703293	LWLT01000021.1-50718645	**0.127**
TLR8		
LWLT01000021.1-50659136	LWLT01000021.1-50659346	**0.118**
LWLT01000021.1-50659136	LWLT01000021.1-50659559	**0.018**
LWLT01000021.1-50659136	LWLT01000021.1-50664064	**0.156**
LWLT01000021.1-50659136	LWLT01000021.1-50664208	**0.156**
LWLT01000021.1-50659136	LWLT01000021.1-50664682	**0.001**
LWLT01000021.1-50659136	LWLT01000021.1-50666071	**0.156**
LWLT01000021.1-50659346	LWLT01000021.1-50659559	0.251
LWLT01000021.1-50659346	LWLT01000021.1-50664064	0.762
LWLT01000021.1-50659346	LWLT01000021.1-50664208	0.762
LWLT01000021.1-50659346	LWLT01000021.1-50664682	**0.008**
LWLT01000021.1-50659346	LWLT01000021.1-50666071	0.762
LWLT01000021.1-50659559	LWLT01000021.1-50664064	0.33
LWLT01000021.1-50659559	LWLT01000021.1-50664208	0.33
LWLT01000021.1-50659559	LWLT01000021.1-50664682	**0.022**
LWLT01000021.1-50659559	LWLT01000021.1-50666071	0.33
LWLT01000021.1-50664064	LWLT01000021.1-50664208	1.0
LWLT01000021.1-50664064	LWLT01000021.1-50664682	**0.01**
LWLT01000021.1-50664064	LWLT01000021.1-50666071	1.0
LWLT01000021.1-50664208	LWLT01000021.1-50664682	**0.01**
LWLT01000021.1-50664208	LWLT01000021.1-50666071	1.0
LWLT01000021.1-50664682	LWLT01000021.1-50666071	**0.01**

r^2 value - the square of the correlation coefficient between two SNPs
